# Mechanotransduction-related ferroptosis: Enhancing boron neutron capture therapy efficacy in glioblastoma using a spheroid model

**DOI:** 10.1016/j.omton.2025.201033

**Published:** 2025-08-09

**Authors:** Lin-Sheng Yu, Jia-Jun Liu, Ming-Hung Yang, Yu-chun Lin, Chi-Shuo Chen

**Affiliations:** 1 Department of Biomedical Engineering and Environmental Sciences, National Tsing Hua University, Hsinchu 30013, Taiwan; 2 Institute of Medical Device and Imaging, School of Medicine, National Taiwan University, Taipei 106319, Taiwan

**Keywords:** MT: Regular Issue, glioblastoma, BNCT, ferroptosis, 3D model

## Abstract

Boron neutron capture therapy (BNCT) shows potential for the treatment of glioblastoma, the most aggressive form of primary brain tumor. Recently, ferroptosis, a cell death triggered by phospholipid peroxidation, has been identified as an important process in tumor therapy. However, the ferroptosis in BNCT has not been fully explored. To investigate ferroptosis induced by BNCT, U87 and U251 human glioblastoma cell lines were used. The results demonstrated that BNCT led to a significant 3- to 4-fold increase in lipid peroxides and caused approximately 25% cell death through ferroptosis in a three-dimensional (3D) spheroid model, which was employed to reconstruct the cell force interactions. Furthermore, BNCT promoted the upregulation of FACL4, an essential protein that triggers ferroptosis and induces oxidative stress by disrupting the endoplasmic reticulum and mitochondria, respectively. Moreover, we found that the mechanosensitive protein YAP-1, known to facilitate ferroptosis, was upregulated and redistributed within the spheroids after BNCT treatment, and the BNCT-induced ferroptosis was enhanced by 1.5-fold following the pharmacological interruption of cell force. This study represents the first demonstration of ferroptosis in BNCT and showed the influences of mechanotransduction in the regulation of ferroptosis, offering a potential strategy to enhance the efficacy of BNCT from the perspective of cell mechanics.

## Graphical abstract

**Figure:**
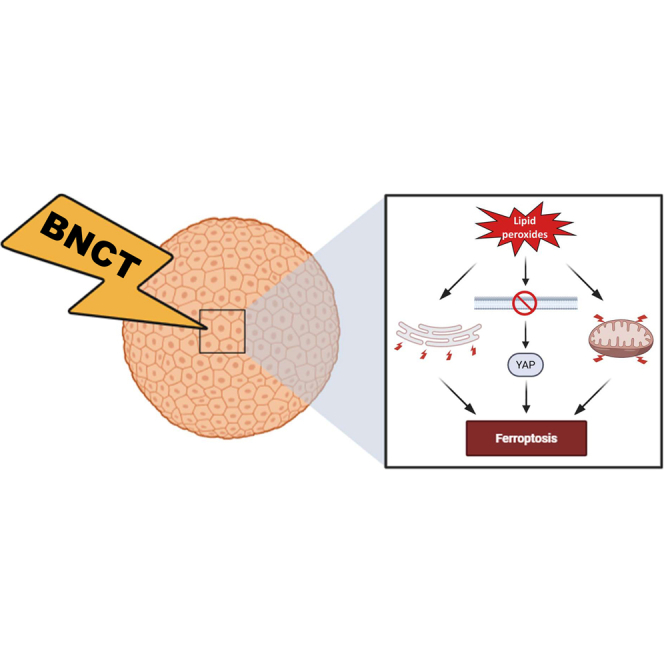

## Teaser

In this study, Chen and colleagues demonstrated BNCT-induced ferroptosis in glioma, and the 3D model showed the influences of mechanotransduction in BNCT-induced ferroptosis. Further, BNCT was enhanced by interrupting the mechanical force. This study is the first to highlight ferroptosis in BNCT and provided insight to improve BNCT with mechanotransduction approach.

## Introduction

Glioblastoma is the most common malignant brain tumor.^1^ After therapy, high-grade glioblastomas are characterized by a median survival of 12–18 months, indicating poor prognosis.^2^ Recently, ferroptosis was revealed, and it was regarded as critical mechanism against glioblastoma.^3^ Different from apoptosis, ferroptosis indicates a cell death initiated by phospholipid peroxidation, which breaks the integrity of cell membrane.^4^ Various studies such as nanozymes for chemodynamic therapy (CDT) and photodynamic therapy (PDT) were developed for glioblastoma, based on the mechanisms of ferroptosis.^5^^,^^6^ Though, BNCT is clinically considered a promising treatment for glioblastoma patients. However, the role of ferroptosis in BNCT remains unclear. In this study, we focused on describing the nature of ferroptosis in BNCT and demonstrating the mechanism of ferroptosis induced by BNCT.

BNCT can effectively prolong the survival in recurrent glioblastoma patients.^7^ The ^10^B(n,α)^7^Li capture reaction can generate α particles, which show the high linear energy transfer (LET) property and the recoiling lithium-7 (^7^Li) atoms. The destructive effect of α particles within boron-containing cells is limited by their small area of action (5–9 μm).^8^ BNCT has been utilized to treat recurrent glioblastomas for around 30 years, and some patients even survived for >10 years.^9^ Recently, clinical data have shown that BNCT was effective in increasing the median survival duration from 14.1 months to 23.5 months in glioblastoma multiforme patients after surgical resection.^10^ Current evidence demonstrates the potential of BNCT in treating glioblastoma. However, detailed mechanism of BNCT in glioblastoma is still unclear.

Mechanical forces have been reported to contribute to glioblastoma tumorigenesis, metastasis, and drug resistance.^11^^,^^12^ Moreover, mechanical forces also regulate metabolism to shape the tumor milieu, and dysregulation of lipid metabolism resulted in inhibition of the therapeutic effect.^13^^,^^14^^,^^15^ These studies show the critical role of mechanical forces in the treatment of glioblastoma. Herein, this study utilized a three-dimensional (3D) spheroid, which is endowed with sufficient mechanical force, as a model and compared both radioresistant and radiosensitive glioblastoma cell lines U87 and U251, respectively, for assessing BNCT-induced ferroptosis. First, we demonstrated how BNCT facilitates cellular lipid peroxidation, which was dysregulated with endoplasmic reticulum (ER) stress and oxidative stress, to trigger ferroptosis in U87 spheroids. Furthermore, the mechanical force contributed to BNCT-induced ferroptosis and subsequently enhanced ferroptosis by Y-27632, a myosin inhibitor. This study provides insights into how BNCT induces ferroptosis in glioblastoma and further demonstrates the interruption of mechanical force to enhance BNCT treatment.

## Results

### Cellular lipids underwent peroxidation after BNCT irradiation

**Figure 1:**
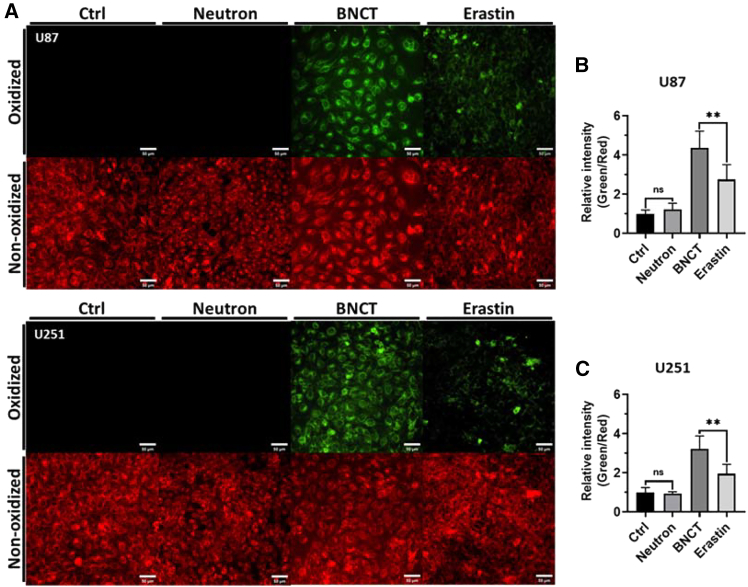
BNCT-induced lipid peroxidation (A) Representative epi-fluorescence images of U87 and U251 cells within the lipid peroxidation probe BDP 581/591 C11. The green signal indicated oxidized lipids, and the red signal indicated non-oxidized lipids. (B and C) represented quantitative results of lipid peroxidation in U87 and U251 cells. All quantitative results were normalized to control group and represented as mean ± SD. Ctrl: without any treatment; Neutron: pure neutron irradiation; BNCT: BNCT treatment; Erastin: 5 μM Erastin treatment. Scale bars, 50 μm. Statistical significance, compared to the control, was determined by t test. ns, not significant. ∗∗∗*p* < 0.001.

The peroxidation process of lipid serves essential role in regulating the stress responses of cell.^16^ Excessive lipid peroxides leading to the destruction of the cell membrane is also a key result of ferroptosis.^17^ Considering the high potency to induce lipid peroxidation of the high-LET alpha particle compared to other LET radiation,^18^ and the lipid-rich content of glioblastoma, we evaluated the alteration of lipid peroxides after BNCT. The lipid peroxidation probe was used for detecting non-oxidized lipid (red) and cellular lipid peroxides (green) (Figure 1A). Following BNCT, the lipid peroxides increased 4- and 2.5-fold in U87 and U251 cells, respectively, but not neutron irradiation (Figures 1B and 1C). BNCT-induced lipid peroxidation was higher than that induced by erastin (5 μM),^19^ a ferroptosis inducer that increased lipid peroxidation approximately 2.5- and 2-fold in U87 and U251 cells, respectively (Figures 1B and 1C). Thus, BNCT was capable of lipid oxidation, illustrating that U87 might be more sensitive to BNCT-induced lipid peroxidation and resulting in ferroptosis.

### BNCT promoted ferroptosis in U87 and U251 glioblastoma spheroids

**Figure 2:**
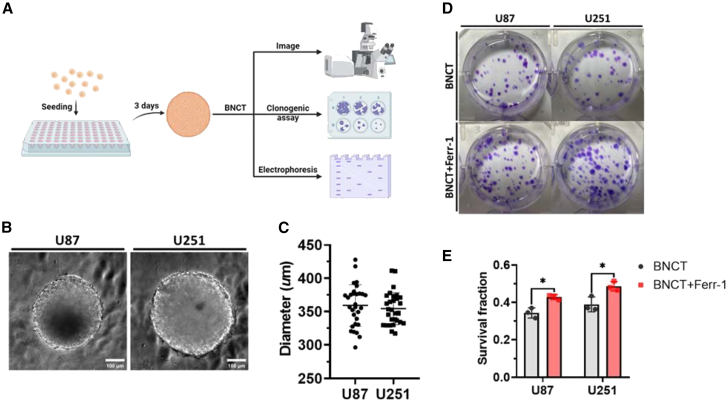
BNCT triggered ferroptosis in glioblastoma spheroids (A) The schematic diagram of the process of an *in vitro* spherical model formation and further experiments. (B) The bright-field images of U87 and U251 spheroids (20× magnification). (C) The quantitative results of the spherical diameter. (D) The photos of the clonogenic assay. (E) The results regarding the survival fraction. All statistical data are represented as mean ± SD. Scale bars, 100 μm. Statistical significance was determined by the t test. ∗*p* < 0.05.

We developed 3D spheroid models of glioblastomas to mimic an *in vivo* solid tumor for evaluating the ferroptosis related to BNCT (Figure 2A).^20^ All spheroids of U87 and U251 cells were cultured for 3 days, with their average diameter reaching approximately 350–370 μm (Figures 2B and 2C). Subsequently, we utilized a clonogenic assay to compare the cell viability with or without 10 μM ferrostatin-1, a ferroptosis inhibitor (Figure 2D). Both spheroids showed an approximately 1.3-fold increase in the survival fraction where ferrostatin-1 was administered, proving the importance of ferroptosis in BNCT treatment. Additionally, BNCT caused a similar cell death, which was close to U251, in U87, the radioresistant glioblastoma, and spheroid (Figure 2E). Thus, these observations demonstrated that BNCT induces ferroptosis with subsequent cell death in the radioresistant glioblastoma spheroid.

### BNCT induced FACL4 and caused ER stress

**Figure 3:**
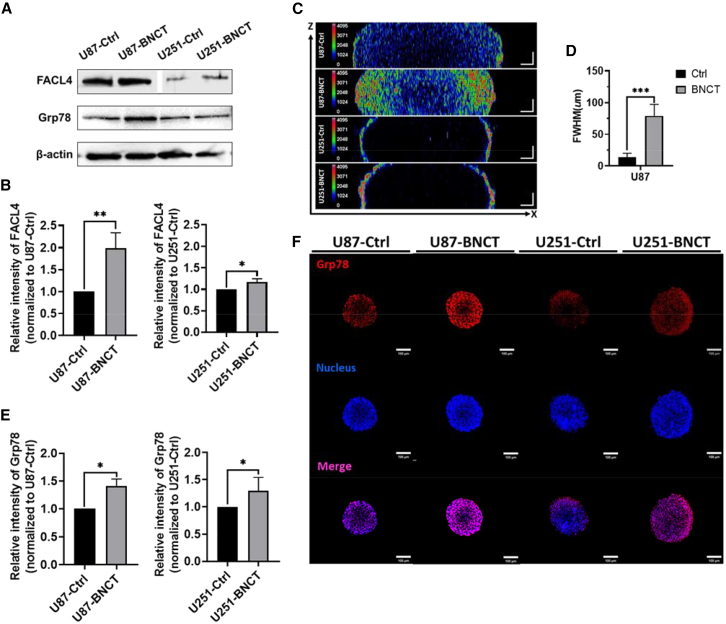
FACL4 changes were mediated by ER stress after BNCT treatment (A) Western blot results. Band of FACL4 was splicing for current arrangement. (B) Quantitative results of western blot for FACL4. (C) The spatial distribution of FACL4. (D) The full width at the half maximum of FACL4 in the U87 spheroid. (E) The quantitative results of immunofluorescent for Grp78. (F) Confocal images of Grp78 immunostaining in glioblastoma spheroids. All spheroids were utilized as control group without BPA and neutron treatment. All statistical data are represented as mean ± SD. Scale bars, 100 μm. 20× magnification. Statistical significance was determined by the t test. ∗*p* < 0.05; ∗∗*p* < 0.01; ∗∗∗*p* < 0.001.

FACL4 was directly upregulated by lipid peroxides and further led to the initiation of ferroptosis.^21^ Due to high level of lipid peroxide accumulation induced by BNCT, we assumed FACL4 would be upregulated after BNCT irradiation. The western blot results indicated an approximately 2-fold upregulation of FACL4 in the U87 spheroid after BNCT in contrast to only 20% upregulation in the U251 spheroid (Figures 3A and 3B). We also identified the spatial distribution of FACL4 over the spheroids and results showed that the full width at half-maximum expression increased to 80 μm, almost six times the value of the control in U87 spheroid (Figures 3C and 3D). The FACL4 prolongation pattern in U87 spheroids supported the amplification of FACL4 by lipid peroxidation^21^ and suggested the potential efficacy of BNCT inside solid tumors. Since FACL4 catalyzed polyunsaturated fatty acids (PUFAs) bound to coenzyme A (CoA) to form PUFA-CoA in the endoplasmic reticulum (ER) oxidation center, upregulation of FACL4 could lead to ER stress.^22^^,^^23^ Glucose regulatory protein 78 (Grp78) is generally regarded as an ER stress sensor, upregulating during ER stress.^24^^,^^25^ We evaluated the variant of Grp78 expression within the BNCT-influenced area (Figure 3A). BNCT significantly induced Grp78 by 40% and 30% in U87 and U251 spheroids, respectively (Figure 3E). Moreover, results of immunofluorescent confocal microscopy demonstrated that the distribution of Grp78 was highly expressed at spherical outer region, which is similar to the pattern of FACL4 (Figures 3C and 3F). Together, BNCT upregulated FACL4 and the following ER stress, further contributing to the ferroptosis in glioblastoma spheroid.

### BNCT interrupted oxidative stress homeostasis through mitochondrial dysregulation

**Figure 4:**
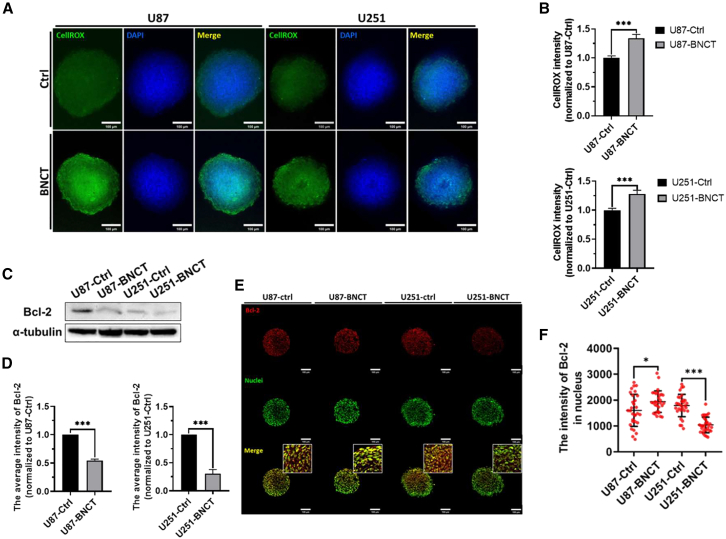
Oxidative stress homeostasis was dysregulated by BNCT (A) A confocal image of ROS (green) and nucleus (blue) in glioblastoma spheroids. (B) The quantitative results of the ROS intensity in U87 and U251 spheroids. (C) Western blot results. (D) The quantitative data for the relative intensity of Bcl-2. (E) A confocal image of Bcl-2 staining in U87 and U251 spheroids. (F) A quantitative Bcl-2 signal inside the nuclei. All statistical data are represented as mean ± SD. Scale bars, 100 μm. 20× magnification. Statistical significance was determined by the t test. ∗*p* < 0.05; ∗∗*p* < 0.01; ∗∗∗*p* < 0.001.

Oxidative stress plays an important role in ferroptosis by producing lipid peroxides. Hence, we monitored reactive oxygen species (ROS) variants inside spheroids after BNCT (Figure 4A). BNCT significantly increased the ROS level by approximately 30% in U87 and U251 spheroids (Figure 4B). Since mitochondrion is an organelle regulating ROS homeostasis, we assessed mitochondrial membrane potential, and the result displayed mitochondrial dysfunction in U87 and U251 cell lines after BNCT irradiation (Figure S1A). Following the administration of BNCT, the ratio of JC-1 fluorescence intensity decreased by approximately 50%, which indicated that BNCT indeed induced mitochondrial dysfunction (Figure S1B). We further evaluated Bcl-2, a well-known ROS-controlling protein residing in the outer mitochondrial membrane, after BNCT.^26^ The expression of Bcl-2 was significantly downregulated by approximately 50% and 70% by BNCT in U87 and U251 spheroids, respectively (Figures 4C and 4D). Interestingly, in contrast to U251, approximately 20% nuclear Bcl-2 signal increased in U87 after BNCT (Figures 4E and 4F). These findings suggest that BNCT might promote Bcl-2 inside nucleus, which interferes the ROS homeostasis in radioresistant glioblastoma.^27^^,^^28^^,^^29^ We suggested that the alteration of Bcl-2 is involved in the dysregulation of oxidative stress induced by BNCT.

### Mechanosensitive protein YAP-1 was activated by BNCT

**Figure 5:**
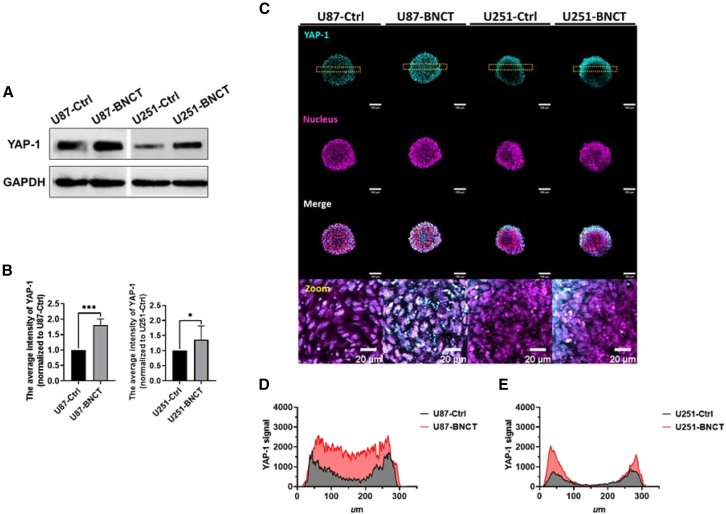
BNCT upregulated YAP-1 and enlarged the region of YAP-1 expression inside the glioblastoma spheroid (A) The pattern of YAP-1 in western blot. Band was splicing for current arrangement. (B) The quantitative results of the average intensity of YAP-1. (C) The confocal images of YAP-1 immunostaining in U87 and U251 spheroids. The red dotted line indicates the area for zoom. (D and E) are the plot profiles of YAP-1 signal in the yellow dotted line area, illustrating YAP-1 distribution inside the spheroids. All statistical data are represented as mean ± SD. Scale bars, 100 μm. 20× magnification. Statistical significance was determined by the t test. ∗*p* < 0.05; ∗∗∗*p* < 0.001.

YAP-1 is a central transcription factor to upregulate ferroptosis,^30^ and YAP-1 is inhibited under the integral cell-cell interaction.^31^ Using a 3D model rich in cellular interactions, we examined the role of YAP-1 in ferroptosis induced by BNCT. Figures 5A and 5B illustrate that YAP-1 was upregulated by 60% and 40% in U87 and U251 spheroids, respectively, by BNCT. In confocal images, BNCT promoted YAP-1 expression in the core of U87 spheroid. Additionally, the plot profile results indicated that the average intensity of gray value increased 1.5- to 2-fold in the U87 spheroid (Figures 5C and 5D). Compared to the control, BNCT extended the area of the YAP-1 expression in the U251 spheroid, and the maximum intensity increased approximately 2-fold (Figures 5C and 5E). Thus, we concluded the upregulation of YAP-1 after BNCT, which may contribute to BNCT-induced ferroptosis.

### Y-27632 enhanced BNCT-induced ferroptosis by interrupting myosin contractility

**Figure 6:**
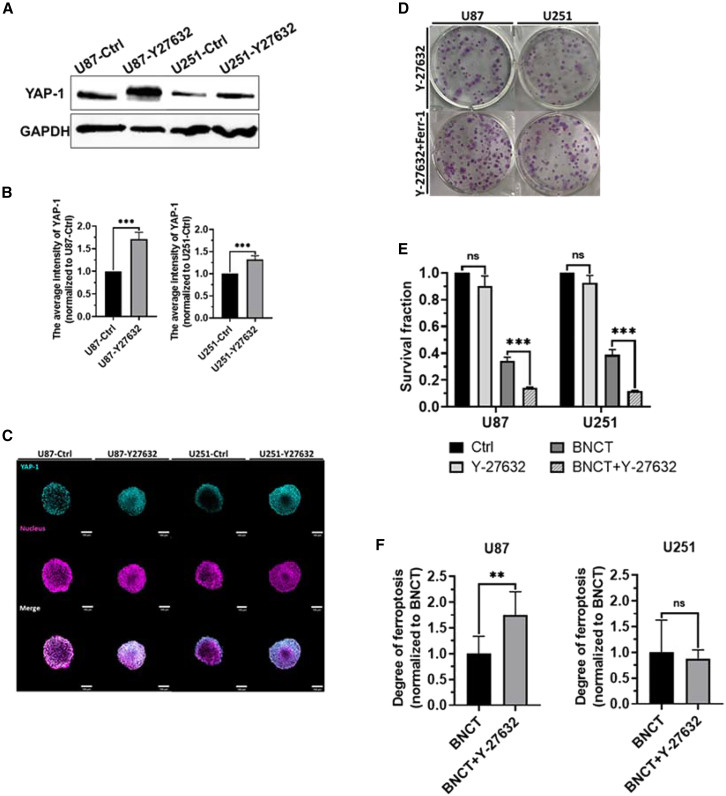
Y-27632 enhances BNCT-induced ferroptosis by increasing YAP-1 expression (A) The pattern of YAP-1 in western blot staining. (B) The quantitative results of the average intensity of YAP-1. (C) Confocal images of YAP-1 distribution inside the glioblastoma spheroids. (D) The photos of the clonogenic assay. (E) The survival fraction of U87 and U251 spheroids within the Y-27632-treated area after BNCT. (F) The level of ferroptosis after ferroptosis inhibitor treatment compared with BNCT alone. All statistical data are represented as mean ± SD. Scale bar, 100 μm. 20× magnification. Statistical significance was determined by the t test. ns, not significant. ∗*p* < 0.05; ∗∗*p* < 0.01; ∗∗∗*p* < 0.001.

The activity of YAP-1 is modulated by mechanical force generated from actin-myosin contractility.^12^^,^^31^ Y-27632, an myosin inhibiter, was used as an adjunctive to increase YAP-1 expression by blocking mechanical force in 3D spheroid, in order to further potentiate ferroptosis induced by BNCT. Our western blot showed that Y27632 does indeed induce a heightened expression of YAP-1 (Figure 6A), 1.7- and 1.3-fold in U87 and U251 spheroids, respectively, by 10 μM Y-27632 treatment^32^ (Figure 6B). Furthermore, immunofluorescent images showed a larger YAP-1 expression area within spheroid after Y-27632 treatment (Figure 6C), confirming that the interrupting of the cell-cell force interaction indeed activated YAP-1 in glioblastoma spheroids. Subsequently, the clonogenic results illustrated that the survival fraction of both U87 and U251 spheroids was reduced approximately by 70% by combining Y-27632 and BNCT compared to BNCT irradiation alone (Figures 6D and 6E). Moreover, ferroptosis induced by BNCT was increased 1.7-fold by adding Y-27632 to the U87 spheroid, although U251 did not increase (Figure 6F). Therefore, pharmaceutically interrupting cell-cell force interaction dramatically enhanced the BNCT effect and even increased the level of ferroptosis in radioresistant glioblastoma.

## Discussion

Although the promising efficacy of BNCT on glioblastoma was clinically demonstrated, the underlying mechanisms remain largely underexplored.^33^ Previous studies have shown that high lipid content U87, rather than U251 cells, was endowed radioresistant against γ-ray,^34^^,^^35^^,^^36^ and we noticed that the susceptibility of U87 cells, known as radioresistant glioblastoma, was close to that of U251 cells after BNCT (Figure 2E). Due to the high LET of alpha particle generated in BNCT process,^37^ high levels of lipid peroxidation were observed in U87 cells after BNCT irradiation.^38^ This finding into previous studies suggests that high level of lipid peroxides induced by BNCT for high lipid-containing cell line, and implied BNCT serves a potential strategy against cancer cells with a lipid-driven therapeutic resistance such as pancreatic adenocarcinoma and breast cancer.^39^^,^^40^

Lipid peroxidation disrupts cellular metabolism and membrane integrity.^41^ We noticed that BNCT upregulated FACL4, indicating the variant of lipid metabolism.^42^ U87 had been considered to be a high lipid-containing cell with high level of FACL4 in regulation of lipid metabolism.^35^^,^^43^ Our results of western blot consisted with previous findings and showed higher FACL4 expression in U87 after BNCT, rather than in U251 (Figure 3A). Though FACL4 is considered as an important regulator in ferroptosis,^17^ no significant BNCT-induced ferroptosis was found between U87 and U251 (Figure 2E). The discrepancy may be owing to the multicausality of ferroptosis, which may be regulated by redox dysregulation of mitochondria or ER stress.^44^ Though FACL4 was described as a role in regulating cell death via modulating lipid metabolism when suffering stress,^45^ the indistinct FACL4 presented in U251 spheroid suggested the other oxidative stress (Figures 4A and 4B) may also lead to the BNCT ferroptosis.^46^ High Grp78, an ER stress marker, also suggested the influence of BNCT on ER regulation, contributing to ferroptosis in glioma.^47^ Our data suggested that higher lipid peroxidation and induced ferroptosis are responsible for the BNCT effect on lipid-rich tissue, such as U87 glioblastoma.

Lipid peroxidation may be directly initiated by the high level of LET alpha particles and stimulated by excessive intracellular ROS.^48^^,^^49^ We evaluated the expression of Bcl-2, which plays a critical role in intracellular ROS regulation.^50^ After BNCT, downregulated Bcl-2 and Bcl-2 translocation to the nucleus suggested the alteration in intracellular ROS homeostasis control by mitochondria.^26^ Compared to a 3D spheroid exposed to X-ray, the ROS-absence regions caused by the shielding of the outer cellular layers lead to undesired tumor survival.^51^^,^^52^ Our cellROX data confirmed the upregulation of intracellular ROS in both U87 and U251 spheroids.^53^ The results suggested that BNCT could stimulate ROS deeply into the solid tumor and potentially reduce the recurrence of “cold tumors.”^54^

As an important mitochondrial protein in the regulation of ROS, the alteration of Bcl-2 also indicated functional changes in mitochondria.^50^ Excessive ROS, such as HO• and H•, generated from the radiolysis of water molecules during BNCT,^55^ might cause the malfunction of mitochondria.^56^ U251 spheroid, endowed with less Bcl-2 protein, expresses similarly ROS signal to U87 (Figures 4A and 4B). The dysregulation of mitochondria may be contributed to by the direct impact of BNCT, which might directly damage mitochondria through the ^10^B(n,α)^7^Li capture reaction inside ^10^B-containing mitochondria.^57^ The results of ROS within U251 spheroid also supported the multiple causes of BNCT-induced ferroptosis, even under lower FACL4 expression (Figures 3A and 3B).^57^^,^^58^ Interestingly, though the overall Bcl-2 expression was decreased, the nuclear Bcl-2 signal in U87 was increased approximately 20% after BNCT (Figures 4E and 4F). Nuclear Bcl-2 has been previously described as inhibiting p53 signaling and suppressing the cellular response to DNA damage by regulating DNA repair mechanisms; our findings aligned with previous findings that U87 cells are more resistant to therapy through apoptosis.^59^^,^^60^^,^^61^ On the other hand, in addition to reducing Bcl-2 expression, the nuclear Bcl-2 suggested the alteration of the ROS homeostasis, which potentially contributes to the ferroptosis responses in radioresistant glioblastoma.^27^^,^^28^^,^^29^ Herein, we underscored the potency of high-LET radiation in BNCT by inducing both apoptosis and ferroptosis, especially in the therapeutically resistant glioblastoma with high lipid contents. Nevertheless, further detailed studies are necessary to uncover the underlying mechanisms driving these effects.

Mechanotransduction substantially influence the effects of glioma treatment.^62^ The reducing intercellular mechanical force would upregulate the YAP-1 expression.^63^^,^^64^ Considering the oxidation of lipids altered the stability of cell membrane,^35^^,^^41^ which might reduce intercellular force transmission.^65^^,^^66^ The observed increase in YAP-1 expression, particularly in U87 spheroids (Figure 5B), might be caused by the alteration of cell membrane integrity after BNCT.^67^ In addition, the increasing of FACL4 expression (Figure 3C) might come from upregulation of YAP-1 expression (Figures 5C and 5D), further inducing ferroptosis.^68^ Furthermore, since YAP-1 was regulated by actin-myosin contractility, utilizing Y-27632, which dysregulates actin dynamics and interrupts the cell-cell contact,^69^ significantly increased YAP-1 expression and dramatically decreased the survival fraction of glioblastoma after BNCT (Figure 6E). However, ferroptosis was only significantly upregulated by Y-27632 treatment after BNCT in the U87 spheroid (Figure 6F).^70^^,^^71^^,^^72^

In current BNCT for glioblastoma treatment, profound efficacy is shown with BPA administered intravenously to cross the blood-brain or blood-tumor barrier (BBB or BTB),^73^ and the ratio of accumulation between tumor and health tissue (T/N ratio or T/B ratio >2.5) is inspected to eliminate the damage of normal tissue nearby before neuron exposure.^74^ Moreover, Y-27632 has been shown to cross BBB protective against neurodegenerative diseases in patients.^75^^,^^76^ Based on the promising clinical outcomes reported in studies, the mechanism described is likely to be clinically applicable. Recently, owing to the development of accelerator-based neutron sources, BNCT is considered to be broadly applied for glioblastoma treatments.^7^ We demonstrated the improved efficacy of BNCT by inhibiting myosin contractility using the FDA-approved compound Y-27632. Additionally, we highlighted the potential of Y-27632 as an adjuvant therapy in BNCT for glioblastoma treatment. However, this hypothesis still needs confirmation through preclinical studies.

### Conclusion

In this study, we demonstrated that BNCT induced lipid peroxidation and ferroptosis in glioma. Furthermore, increased ER stress and intracellular ROS contributed to BNCT-induced ferroptosis. Additionally, utilizing the 3D model, we investigated the influences of BNCT-activated mechanotransduction signaling YAP on the ferroptosis. We further showed the enhanced effectiveness of BNCT by interrupting the myosin contractility with a Food and Drug Administration (FDA)-approved reagent Y-27632, as well as the potential of Y-27632 as adjuvant therapy for BNCT treatment of glioma. In summary, our study is the first to highlight the importance of ferroptosis in BNCT for glioma treatment and provided insight to reach the higher effectiveness of BNCT in glioblastoma treatment with a mechanotransduction approach.

## Materials and methods

### Cell culture

Both U87.CD4.CCR5 (CVCL_X630) and U-251MG (CVCL_0021), which derived from a male patient, are human glioblastoma cell lines, obtained from the Bioresource Collection and Research Center (Hsinchu, Taiwan). U87.CD4.CCR5 (CVCL_X630) was seen as radioresistant. Otherwise, U-251MG (CVCL_0021) was regarded sensitive to radiotherapy.^60^ Cells were maintained with high-glucose Dulbecco’s modified Eagle medium (Thermo Fisher Scientific, California, USA), which was supplemented with 10% inactive fetal bovine serum and 100 U/mL penicillin-streptomycin (Thermo Fisher Scientific, California, USA).

### 3D glioblastoma spheroid formation and morphology

We produced 3D glioblastoma spheroid using 96-well U-bottom plates precoated with 0.7% Pluronic F-127 (Sigma-Aldrich, Taufkirchen, Germany) to produce low cell-contact surface. Then, U87 and U251 cells were seeded in 96-well plates (1 × 10^4^ cells/well). We changed medium every 2 days. We utilized a Hamamatsu ORCA-flash 4.0 camera and Nikon ECLIPSE Ti microscopy to capture images on day 3. All spheroids were assigned to experimental groups by simple randomization. Later, the images analysis were performed using software ImageJ (RRID:SCR_003070).

### BNCT

Spheroids were treated with 520 μg/mL boronophenylalanine (BPA) (25 μg/mL ^10^B) for 4 h to accumulate ^10^B and irradiated with BNCT subsequently. The neutrons source is from Tsing Hua Open-Pool Reactor (THOR, National Tsing Hua University, Taiwan). The irradiation condition was 1.2 MW for 8 min, with 6 × 10^7^ cm^−2^s^−1^ thermal neutron and 8.2 × 10^8^ cm^−2^s^−1^ epithermal neutron.

### Lipid peroxide assessment

U87 and U251 cells were seeded and treated with BNCT irradiation. Later, all cells were washed by PBS and stained with a lipid peroxidation probe BDP 581/591 C11 (Dojindo Laboratories, Japan) 50 μM for 30 min. The red fluorescent would turn to green when reacted with lipid radicals. Neutron-alone irradiation and erastin treatment were utilized as negative and positive control, respectively. Control represented sample without any treatment. The fluorescent signal was evaluated by a Hamamatsu ORCA-flash 4.0 camera and Nikon ECLIPSE Ti microscopy. For calculating the degree of lipid peroxidation, the green intensity is divided by red intensity as final result.

### Clonogenic assay

For ferrostatin-1 treatment group, all spheroids were treated with 10 μM ferrostatin-1 before BNCT irradiation and continually changed ferrostatin-1 every 2 days until colonies formation. U87 and U251 spheroids were separated into single-cell suspension using stemPro accutase dissociation solution (Thermo Fisher Scientific, California, USA). Subsequently, cells were seeded and cultured until colony formation. All colonies were fixed with glutaraldehyde (6.0% v/v), stained with crystal violet (0.5% w/v) for 30 min, and counted by a stereomicroscope. The surviving fraction calculation was following this literature.^77^

### ROS assessment

After BNCT for 24 h, U87 and U251 spheroids was stained with CellROX green reagent (Thermo Fisher Scientific, California, USA) for 30 min, subsequently fixed by 4% paraformaldehyde and permeabilized using PBS with 2% Triton X-100. Cell nucleus was labeled through SYTOX Green Nucleic Acid Stain (Thermo Fisher Scientific, California, USA), and RapiClear 1.52 (SunJin Lab, Hsinchu, Taiwan) was utilized for transparency. The fluorescent images were captured by Nikon ECLIPSE Ti microscopy with the CSU-X1 system and the Hamamatsu ORCA-flash 4.0 camera. Then, ImageJ 1.53j software (RRID:SCR_003070) was utilized to analyze all images.

### Mitochondrial membrane potential assessment

After BNCT for 3 h, U87 and U251 cell lines was stained with JC-1 dye (Thermo Fisher Scientific, California, USA) 5 μM for 30 min, subsequently capturing the fluorescent image by a Hamamatsu ORCA-flash 4.0 camera and Nikon ECLIPSE Ti microscopy. For calculating the degree of mitochondria dysregulation, the red intensity is divided by green intensity as finial result.

### Immunofluorescence and confocal microscopy

Spheroids were fixed by 4% paraformaldehyde and permeabilized with 2% Triton X-100. The samples were treated with the corresponding primary antibodies overnight at 4°C, following the secondary antibodies. All application for antibodies was described in supplemental (Table S1). Cell nucleus was labeled through SYTOX Green Nucleic Acid Stain (Thermo Fisher Scientific, California, USA). The 3D spheroids were treated with RapiClear 1.52 (SunJin Lab, Hsinchu, Taiwan) for transparency. All samples were visualized utilizing a Confocal Laser Scanning Microscope (IX 81, Olympus, Japan).

### Western blot

All steps for samples preparation were following the previous study.^78^ Afterward, all samples were separated by SDS-PAGE as previously described^79^ and incubated with primary antibodies overnight at 4°C. Then, secondary antibodies were applied. Images were exhibited utilizing Trident femto Western HRP Substrate (GTX14698).

### Statistical analysis

The data are presented as mean ± standard deviation (SD) of at least three time repeat experiments. All results were stated by Student’s t test with two tails to test statistical significance, and a *p* value <0.05 indicated statistical significance. We performed the analysis using GraphPad 8.0 (RRID:SCR_002798).

## Data availability

All data supporting the findings of present study are available within the article and its supplemental.

## Acknowledgments

The authors acknowledge for funding from National Science and Technology Council (NSTC), NSTC- 113-2112-M-007 -025 -; NSTC-113-2923-M-007 -003 -MY2; NSTC-113-2635-E-002 -001 -; and National Tsing Hua Univeristy, NTHU-114Q2725E1.

## Author contributions

L.-S.Y., conceptualization, data curation, formal analysis, investigation, methodology, project administration, software, validation, visualization, writing—original draft, and writing—review & editing. J.-J.L., methodology and validation. M.-H.Y., methodology and validation. Y.-C.L., funding acquisition and resources. C.-S.C., conceptualization, funding acquisition, investigation, project administration, resources, supervision, writing—original draft, and writing—review & editing.

## Declaration of interests

The authors declare no competing interests.

## Supplemental information

Document S1. Figure S1 and Table S1

Document S2. Article plus supplemental information

## References

[1] Ostrom Q.T., Cioffi G., Gittleman H., Patil N., Waite K., Kruchko C., Barnholtz-Sloan J.S. (2019). Cbtrus statistical report: Primary brain and other central nervous system tumors diagnosed in the united states in 2012–2016. Neuro Oncol..

[2] Wen P.Y., Kesari S. (2008). Malignant gliomas in adults. N. Engl. J. Med..

[3] Zhuo S., He G., Chen T., Li X., Liang Y., Wu W., Weng L., Feng J., Gao Z., Yang K. (2022). Emerging role of ferroptosis in glioblastoma: Therapeutic opportunities and challenges. Front. Mol. Biosci..

[4] Jiang X., Stockwell B.R., Conrad M. (2021). Ferroptosis: Mechanisms, biology and role in disease. Nat. Rev. Mol. Cell Biol..

[5] Xu L., Hu J., Yan X., Zhao H., Geng M., Zhang J., Zhou C., Liu Z., Li B., Hu S. (2024). Overcoming therapeutic limitations in glioblastoma treatment: A nanocomposite inducing ferroptosis and oxeiptosis via photodynamic therapy. Chem. Eng. J..

[6] Kojima Y., Tanaka M., Sasaki M., Ozeki K., Shimura T., Kubota E., Kataoka H. (2024). Induction of ferroptosis by photodynamic therapy and enhancement of antitumor effect with ferroptosis inducers. J. Gastroenterol..

[7] Kawabata S., Suzuki M., Hirose K., Tanaka H., Kato T., Goto H., Narita Y., Miyatake S.-I. (2021). Accelerator-based bnct for patients with recurrent glioblastoma: A multicenter phase ii study. Neurooncol. Adv..

[8] Jin W.H., Seldon C., Butkus M., Sauerwein W., Giap H.B. (2022). A review of boron neutron capture therapy: Its history and current challenges. Int. J. Part. Ther..

[9] Hatanaka H., Nakagawa Y. (1994). Clinical results of long-surviving brain tumor patients who underwent boron neutron capture therapy. Int. J. Radiat. Oncol. Biol. Phys..

[10] Kawabata S., Miyatake S.I., Kuroiwa T., Yokoyama K., Doi A., Iida K., Miyata S., Nonoguchi N., Michiue H., Takahashi M. (2009). Boron neutron capture therapy for newly diagnosed glioblastoma. J. Radiat. Res..

[11] Bhargav A.G., Domino J.S., Chamoun R., Thomas S.M. (2021). Mechanical properties in the glioma microenvironment: Emerging insights and theranostic opportunities. Front. Oncol..

[12] Aguilar-Cuenca R., Juanes-García A., Vicente-Manzanares M. (2014). Myosin ii in mechanotransduction: Master and commander of cell migration, morphogenesis, and cancer. Cell. Mol. Life Sci..

[13] Bacci M., Lorito N., Smiriglia A., Morandi A. (2021). Fat and furious: Lipid metabolism in antitumoral therapy response and resistance. Trends Cancer.

[14] Kao T.J., Lin C.L., Yang W.B., Li H.Y., Hsu T.I. (2023). Dysregulated lipid metabolism in tmz-resistant glioblastoma: Pathways, proteins, metabolites and therapeutic opportunities. Lipids Health Dis..

[15] Bertolio R., Napoletano F., Del Sal G. (2023). Dynamic links between mechanical forces and metabolism shape the tumor milieu. Curr. Opin. Cell Biol..

[16] Gaschler M.M., Stockwell B.R. (2017). Lipid peroxidation in cell death. Biochem. Biophys. Res. Commun..

[17] Dixon S.J., Olzmann J.A. (2024). The cell biology of ferroptosis. Nat. Rev. Mol. Cell Biol..

[18] Kim E.H., Kim M.S., Lee K.H., Sai S., Jeong Y.K., Koh J.S., Kong C.B. (2017). Effect of low- and high-linear energy transfer radiation on in vitro and orthotopic in vivo models of osteosarcoma by activation of caspase-3 and -9. Int. J. Oncol..

[19] Miao Z., Tian W., Ye Y., Gu W., Bao Z., Xu L., Sun G., Li C., Tu Y., Chao H. (2022). Hsp90 induces acsl4-dependent glioma ferroptosis via dephosphorylating ser637 at drp1. Cell Death Dis..

[20] Zanoni M., Piccinini F., Arienti C., Zamagni A., Santi S., Polico R., Bevilacqua A., Tesei A. (2016). 3d tumor spheroid models for in vitro therapeutic screening: A systematic approach to enhance the biological relevance of data obtained. Sci. Rep..

[21] Zhang H.-L., Hu B.-X., Li Z.-L., Du T., Shan J.-L., Ye Z.-P., Peng X.-D., Li X., Huang Y., Zhu X.-Y. (2022). Pkcβii phosphorylates acsl4 to amplify lipid peroxidation to induce ferroptosis. Nat. Cell Biol..

[22] Zhou R., Wei K., Li X., Yan B., Li L. (2024). Mechanisms of ferroptosis and the relationship between ferroptosis and er stress after jev and hsv infection. Front. Microbiol..

[23] Jakobsen C.H., Størvold G.L., Bremseth H., Follestad T., Sand K., Mack M., Olsen K.S., Lundemo A.G., Iversen J.G., Krokan H.E., Schønberg S.A. (2008). Dha induces er stress and growth arrest in human colon cancer cells: Associations with cholesterol and calcium homeostasis. J. Lipid Res..

[24] Dauer P., Sharma N.S., Gupta V.K., Durden B., Hadad R., Banerjee S., Dudeja V., Saluja A., Banerjee S. (2019). Er stress sensor, glucose regulatory protein 78 (grp78) regulates redox status in pancreatic cancer thereby maintaining “stemness”. Cell Death Dis..

[25] Chen J., Lynn E.G., Yousof T.R., Sharma H., MacDonald M.E., Byun J.H., Shayegan B., Austin R.C. (2022). Scratching the surface—an overview of the roles of cell surface grp78 in cancer. Biomedicines.

[26] Chong S.J.F., Low I.C.C., Pervaiz S. (2014). Mitochondrial ros and involvement of bcl-2 as a mitochondrial ros regulator. Mitochondrion.

[27] Froesch B.A., Aimé-Sempé C., Leber B., Andrews D., Reed J.C. (1999). Inhibition of p53 transcriptional activity by bcl-2 requires its membrane-anchoring domain. J. Biol. Chem..

[28] Hemann M.T., Lowe S.W. (2006). The p53-bcl-2 connection. Cell Death Differ..

[29] Vigneron A., Vousden K.H. (2010). P53, ros and senescence in the control of aging. Aging.

[30] Yang W.H., Lin C.C., Wu J., Chao P.Y., Chen K., Chen P.H., Chi J.T. (2021). The hippo pathway effector yap promotes ferroptosis via the e3 ligase skp2. Mol. Cancer Res..

[31] Cai X., Wang K.-C., Meng Z. (2021). Mechanoregulation of yap and taz in cellular homeostasis and disease progression. Front. Cell Dev. Biol..

[32] Driscoll T.P., Cosgrove B.D., Heo S.-J., Shurden Z.E., Mauck R.L. (2015). Cytoskeletal to nuclear strain transfer regulates yap signaling in mesenchymal stem cells. Biophys. J..

[33] Miyatake S.I., Kawabata S., Hiramatsu R., Kuroiwa T., Suzuki M., Ono K. (2018). Boron neutron capture therapy of malignant gliomas. Prog. Neurol. Surg..

[34] Subbarao R.B., Ok S.H., Lee S.H., Kang D., Kim E.J., Kim J.Y., Sohn J.T. (2018). Lipid emulsion inhibits the late apoptosis/cardiotoxicity induced by doxorubicin in rat cardiomyoblasts. Cells.

[35] Wu X., Geng F., Cheng X., Guo Q., Zhong Y., Cloughesy T.F., Yong W.H., Chakravarti A., Guo D. (2020). Lipid droplets maintain energy homeostasis and glioblastoma growth via autophagic release of stored fatty acids. iScience.

[36] Su Y., Li T. (2024). Radioresistance via lipid metabolism: Intrinsic, acquired, and tumor microenvironment. Precision Nutr..

[37] Nikitaki Z., Velalopoulou A., Zanni V., Tremi I., Havaki S., Kokkoris M., Gorgoulis V.G., Koumenis C., Georgakilas A.G. (2022). Key biological mechanisms involved in high-let radiation therapies with a focus on DNA damage and repair. Expert Rev. Mol. Med..

[38] Agarwal S., Chatterjee S.N. (1984). Peroxidation of the dried thin film of lipid by high-energy alpha particles from a cyclotron. Radiat. Res..

[39] Germain N., Dhayer M., Boileau M., Fovez Q., Kluza J., Marchetti P. (2020). Lipid metabolism and resistance to anticancer treatment. Biology.

[40] Wang Z., Wang Y., Li Z., Xue W., Hu S., Kong X. (2023). Lipid metabolism as a target for cancer drug resistance: Progress and prospects. Front. Pharmacol..

[41] Li J., Kang R., Tang D., Kepp O., Galluzzi L. (2021). Methods in Cell Biology.

[42] Chen J., Ding C., Chen Y., Hu W., Yu C., Peng C., Feng X., Cheng Q., Wu W., Lu Y. (2021). Acsl4 reprograms fatty acid metabolism in hepatocellular carcinoma via c-myc/srebp1 pathway. Cancer Lett..

[43] Hacioglu C., Kar F. (2023). Capsaicin induces redox imbalance and ferroptosis through acsl4/gpx4 signaling pathways in u87-mg and u251 glioblastoma cells. Metab. Brain Dis..

[44] Chen X., Kang R., Kroemer G., Tang D. (2021). Organelle-specific regulation of ferroptosis. Cell Death Differ..

[45] Chen F., Kang R., Liu J., Tang D. (2023). The acsl4 network regulates cell death and autophagy in diseases. Biology.

[46] Yu Y., Yan Y., Niu F., Wang Y., Chen X., Su G., Liu Y., Zhao X., Qian L., Liu P., Xiong Y. (2021). Ferroptosis: A cell death connecting oxidative stress, inflammation and cardiovascular diseases. Cell Death Discov..

[47] Lee Y.S., Lee D.H., Choudry H.A., Bartlett D.L., Lee Y.J. (2018). Ferroptosis-induced endoplasmic reticulum stress: Cross-talk between ferroptosis and apoptosis. Mol. Cancer Res..

[48] Lu M., Zhou Y., Sun L., Shafi S., Ahmad N., Sun M., Dong J. (2022). The molecular mechanisms of ferroptosis and its role in glioma progression and treatment. Front. Oncol..

[49] Su L.J., Zhang J.H., Gomez H., Murugan R., Hong X., Xu D., Jiang F., Peng Z.Y. (2019). Reactive oxygen species-induced lipid peroxidation in apoptosis, autophagy, and ferroptosis. Oxid. Med. Cell. Longev..

[50] Aharoni-Simon M., Shumiatcher R., Yeung A., Shih A.Z.L., Dolinsky V.W., Doucette C.A., Luciani D.S. (2016). Bcl-2 regulates reactive oxygen species signaling and a redox-sensitive mitochondrial proton leak in mouse pancreatic β-cells. Endocrinology.

[51] Choi U.Y., Lee J.J., Park A., Zhu W., Lee H.R., Choi Y.J., Yoo J.S., Yu C., Feng P., Gao S.J. (2020). Oncogenic human herpesvirus hijacks proline metabolism for tumorigenesis. Proc. Natl. Acad. Sci. USA.

[52] Brüningk S.C., Ziegenhein P., Rivens I., Oelfke U., Haar G. (2019). A cellular automaton model for spheroid response to radiation and hyperthermia treatments. Sci. Rep..

[53] Broekgaarden M., Bulin A.-L., Porret E., Musnier B., Chovelon B., Ravelet C., Sancey L., Elleaume H., Hainaut P., Coll J.-L., Le Guével X. (2020). Surface functionalization of gold nanoclusters with arginine: A trade-off between microtumor uptake and radiotherapy enhancement. Nanoscale.

[54] Świerczewska M., Sterzyńska K., Ruciński M., Andrzejewska M., Nowicki M., Januchowski R. (2023). The response and resistance to drugs in ovarian cancer cell lines in 2d monolayers and 3d spheroids. Biomed. Pharmacother..

[55] Zou Z., Chang H., Li H., Wang S. (2017). Induction of reactive oxygen species: An emerging approach for cancer therapy. Apoptosis.

[56] Napolitano G., Fasciolo G., Venditti P. (2021). Mitochondrial management of reactive oxygen species. Antioxidants.

[57] Tien N., Brownell G.L., Holden S.A., Teicher B.A. (1993). Intracellular distribution of various boron compounds for use in boron neutron capture therapy. Biochem. Pharmacol..

[58] Feng H., Stockwell B.R. (2018). Unsolved mysteries: How does lipid peroxidation cause ferroptosis?. PLoS Biol..

[59] Marei H.E., Althani A., Afifi N., Hasan A., Caceci T., Pozzoli G., Morrione A., Giordano A., Cenciarelli C. (2021). P53 signaling in cancer progression and therapy. Cancer Cell Int..

[60] Naidu M.D., Mason J.M., Pica R.V., Fung H., Peña L.A. (2010). Radiation resistance in glioma cells determined by DNA damage repair activity of ape1/ref-1. J. Radiat. Res..

[61] Annovazzi L., Caldera V., Mellai M., Riganti C., Battaglia L., Chirio D., Melcarne A., Schiffer D. (2015). The DNA damage/repair cascade in glioblastoma cell lines after chemotherapeutic agent treatment. Int. J. Oncol..

[62] Crivii C.B., Boşca A.B., Melincovici C.S., Constantin A.M., Mărginean M., Dronca E., Suflețel R., Gonciar D., Bungărdean M., Șovrea A. (2022). Glioblastoma microenvironment and cellular interactions. Cancers.

[63] Dasgupta I., McCollum D. (2019). Control of cellular responses to mechanical cues through yap/taz regulation. J. Biol. Chem..

[64] Li W., Shu X., Zhang X., Zhang Z., Sun S., Li N., Long M. (2023). Potential roles of yap/taz mechanotransduction in spaceflight-induced liver dysfunction. Int. J. Mol. Sci..

[65] Catalá A., Díaz M. (2016). Editorial: Impact of lipid peroxidation on the physiology and pathophysiology of cell membranes. Front. Physiol..

[66] Lim C.G., Jang J., Kim C. (2018). Cellular machinery for sensing mechanical force. BMB Rep..

[67] Ammendolia D.A., Bement W.M., Brumell J.H. (2021). Plasma membrane integrity: Implications for health and disease. BMC Biol..

[68] Sun T., Chi J.-T. (2021). Regulation of ferroptosis in cancer cells by yap/taz and hippo pathways: The therapeutic implications. Genes Dis..

[69] Gao L., Nath S.C., Jiao X., Zhou R., Nishikawa S., Krawetz R., Li X., Rancourt D.E. (2019). Post-passage rock inhibition induces cytoskeletal aberrations and apoptosis in human embryonic stem cells. Stem Cell Res..

[70] Pranatharthi A., Ross C., Srivastava S. (2016). Cancer stem cells and radioresistance: Rho/rock pathway plea attention. Stem Cells Int..

[71] Li H., Yang T., Zhang J., Xue K., Ma X., Yu B., Jin X. (2024). Pyroptotic cell death: An emerging therapeutic opportunity for radiotherapy. Cell Death Discov..

[72] Jiao Y., Cao F., Liu H. (2022). Radiation-induced cell death and its mechanisms. Health Phys..

[73] Shimizu S., Nakai K., Li Y., Mizumoto M., Kumada H., Ishikawa E., Yamamoto T., Matsumura A., Sakurai H. (2023). Boron neutron capture therapy for recurrent glioblastoma multiforme: Imaging evaluation of a case with long-term local control and survival. Cureus.

[74] Tokura D., Konarita K., Suzuki M., Ogata K., Honda Y., Miura Y., Nishiyama N., Nomoto T. (2024). Active control of pharmacokinetics using light-responsive polymer-drug conjugates for boron neutron capture therapy. J. Control. Release.

[75] Koch J.C., Tatenhorst L., Roser A.-E., Saal K.-A., Tönges L., Lingor P. (2018). Rock inhibition in models of neurodegeneration and its potential for clinical translation. Pharmacol. Ther..

[76] Jeon B.T., Jeong E.A., Park S.Y., Son H., Shin H.J., Lee D.H., Kim H.J., Kang S.S., Cho G.J., Choi W.S., Roh G.S. (2013). The rho-kinase (rock) inhibitor y-27632 protects against excitotoxicity-induced neuronal death in vivo and in vitro. Neurotox. Res..

[77] Franken N.A.P., Rodermond H.M., Stap J., Haveman J., van Bree C. (2006). Clonogenic assay of cells in vitro. Nat. Protoc..

[78] Yu L.S., Jhunjhunwala M., Hong S.Y., Yu L.Y., Lin W.R., Chen C.S. (2021). Tissue architecture influences the biological effectiveness of boron neutron capture therapy in in vitro/in silico three-dimensional self-assembly cell models of pancreatic cancers. Cancers.

[79] Mahmood T., Yang P.C. (2012). Western blot: Technique, theory, and trouble shooting. N. Am. J. Med. Sci..

