# Supplementary material for: Mechanotransduction-related ferroptosis: Enhancing boron neutron capture therapy efficacy in glioblastoma using a spheroid model
Source: Mol Ther Oncol. 2025 Aug 9;33(3):201033. doi: 10.1016/j.omton.2025.201033 (PMC12398792; doi:10.1016/j.omton.2025.201033)
Supplement: Document S1. Figure S1 and Table S1 [file mmc1.pdf]

OMTON, Volume *33*

Supplemental information

Mechanotransduction-related ferroptosis: Enhancing boron neutron capture therapy efficacy in glioblastoma using a spheroid model

Lin-Sheng Yu, Jia-Jun Liu, Ming-Hung Yang, Yu-chun Lin, and Chi-Shuo Chen

Supplemental

Table S1. The antibodies application in present research

	targets	conjugated fluorophores	clones	working dilutions	manufacturers
FACL4<br>RRID:AB_11003294	Human, Mouse, Rat		Polyclonal	IF:1:200<br>WB:1:3,000	GeneTex<br>GTX100260
Grp78<br>RRID:AB_597753	Human, Mouse, Rat, Chicken		Polyclonal	IF:1:200<br>WB:1:3,000	GeneTex<br>GTX113340
Bcl-2<br>RRID:AB_289591	Human, Mouse, Rat, Cat, Dog		Polyclonal	IF:1:200<br>WB:1:1,000	GeneTex<br>GTX100064
YAP-1<br>RRID:AB_2619554	Human, Mouse, Deer		Polyclonal	IF:1:200<br>WB:1:3,000	GeneTex<br>GTX129151
β-actin<br>RRID:AB_1543252	Human, Mouse, Rat, Rabbit, Goat		Monoclonal	WB:1:10,000	GeneTex<br>GTX629630
α-tubulin<br>RRID:AB_856576	Human, Mouse, Rat, Rabbit, Goat		Monoclonal	WB:1:10,000	GeneTex<br>GTX628802
GAPDH<br>RRID:AB_1067086	Human, Mouse, Rat, Rabbit, Goat		Polyclonal	WB:1:10,000	GeneTex<br>GTX100118
AffiniPure™ Goat Anti-Rabbit IgG (H+L)<br>RRID:AB_2338059	Rabbit	Alexa Fluor® 594	Polyclonal	1:800	Jackson ImmunoResearch Inc<br>Code: 111-005-003
Goat Anti-Rabbit IgG antibody<br>RRID:AB_2687483	Rabbit	Horseradish peroxidase	Polyclonal	1:10,000	GeneTex<br>GTX213110-01
AffiniPure™ Goat Anti-Mouse IgG (H+L)<br>RRID:AB_10015289	Mouse	Horseradish peroxidase	Polyclonal	1:10,000	Jackson ImmunoResearch Inc<br>Code: 115-005-003

Supplemental

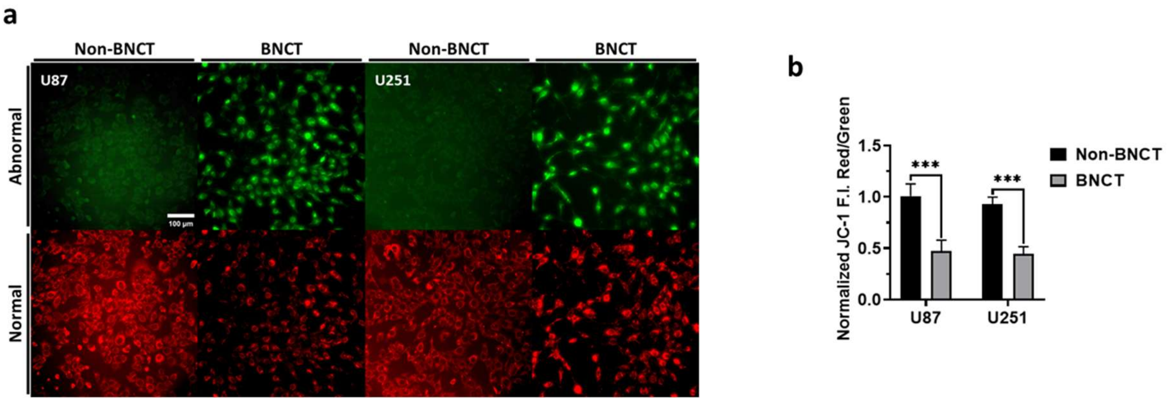

Figure S1. BNCT induced mitochondrial stress. (a) represented mitochondrial stress indicator JC-1 staining. Green: abnormal mitochondria. Red: normal mitochondria. 20× Magnification. (b) the quantitative result of JC-1 staining. All statistical data are represented as mean ± SD. Scale bar = 100 μm. 20× Magnification. Statistical significance was determined by the t-test. ***, $p < 0.001$.
